# The global landscape of bladder cancer incidence and mortality in 2020 and projections to 2040

**DOI:** 10.7189/jogh.13.04109

**Published:** 2023-09-15

**Authors:** Yanting Zhang, Harriet Rumgay, Mengmeng Li, Haibing Yu, Haiyan Pan, Jindong Ni

**Affiliations:** 1 Department of Epidemiology and Health Statistics, School of Public Health, Guangdong Medical University, Dongguan, China; 2 Cancer Surveillance Branch, International Agency for Research on Cancer, Lyon, France; 3 Department of Cancer Prevention, State Key Laboratory of Oncology in South China, Collaborative Innovation Center for Cancer Medicine, Sun Yat-sen University Cancer Center, Guangzhou, China

## Abstract

**Background:**

Bladder cancer (BCa) is one of the most common urological malignancies worldwide. This study examines the global epidemiological profile of BCa incidence and mortality in 2020 and the projected burden to 2040.

**Methods:**

The estimated number of BCa cases and deaths were extracted from the GLOBOCAN 2020 database. Age-standardised incidence rates (ASIRs) and age-standardised mortality rates (ASMRs) were calculated using the world standard. The predicted BCa incidence and mortality in 2040 was calculated based on demographic projections.

**Results:**

Globally, approximately 573 000 new BCa cases and 213 000 deaths occurred in 2020, corresponding to ASIRs and ASMRs of 5.6 and 1.9 per 100 000, respectively. The incidence and mortality rates were approximately 4-fold higher in men (9.5 and 3.3 per 100 000, respectively) than women (2.4 and 0.9, respectively). Across world regions, incidence rates varied at least 12-fold among men and 8-fold among women, with the highest ASIRs for both men and women detected in Southern Europe (26.5 and 5.8 per 100 000, respectively) and Western Europe (21.5 and 5.8, respectively) and the lowest in Middle Africa (2.2) in men and South-Central Asia (0.7) in women. The highest ASMRs for both men and women were found in Northern Africa (9.2 and 1.8 per 100 000, respectively). By 2040, the annual number of new BCa cases and deaths will increase to 991 000 (72.8% increase from 2020) and 397 000 (86.6% increase), respectively.

**Conclusions:**

Geographical distributions of BCa incidence and mortality uncovered higher risk of BCa incidence in Southern and Western European populations and higher risk of mortality in Northern African populations. Considering the predicted 73% and 87% increase in annual BCa cases and deaths by 2040 globally, respectively, there is an urgent need to develop and accelerate BCa control initiatives for high-risk populations to tackle global BCa burden and narrow its geographical disparities.

Bladder cancer (BCa) is the 10^th^ most commonly diagnosed cancer and the thirteenth leading cause of cancer death worldwide [1,2]. A previous study showed that more than 60% of all BCa cases and about half of its deaths were observed in less developed countries in 2012 [3]. However, over the past decade, incidence of BCa in Europe has increased while mortality has decreased, whereas in Asia BCa incidence has decreased but mortality from BCa in men has increased [4]. Notably, distinct sex disparities were found for BCa, with three-quarters of cases occurring in men in 2012 [3]. Furthermore, compared to other urological cancers, 5-year survival from BCa has remained relatively low and there are considerable inequalities in survival e.g., 77% in Peru and 34% in Colombia [5,6].

BCa is a largely preventable disease due to its many modifiable risk factors. Tobacco smoking is the principal risk factor for BCa, accounting for about half of all BCa cases [7-9] and 37% of its deaths [10]. Current and former smokers are considered to have a 3.5- and 2.0-fold increased risk of BCa compared with nonsmokers, respectively [11]. The second greatest risk factor for BCa after smoking is occupational exposure to carcinogens [12]. Approximately 10% of all BCa cases can be attributed to occupational exposure to carcinogenic chemicals, such as aromatic amines, polycyclic aromatic hydrocarbons, and chlorinated hydrocarbons [12-14]. Another important risk factor for BCa is *Schistosoma haematobium* infection [15,16].

Given the recent changes in geographical disparities in the burden of BCa [3,4] and the strong association of BCa with its modifiable risk factors, understanding the current epidemiological profile of international variations in BCa incidence and mortality is important. This would allow policymakers to make evidence-based decisions for primary prevention and to optimise the allocation of resources to reduce the global burden of BCa. Moreover, BCa is more likely to affect older people [17], and considering the growing and ageing global population, predicting the future BCa burden is essential for cancer control planning. Although previous studies have reported the burden of BCa incidence and mortality in 2012 [3] and the changing epidemiological profiles of BCa burden over the past decades [4], recent and comprehensive epidemiological data on BCa incidence and mortality and quantitative projections of the future BCa burden are still lacking.

Herein, we aimed to better understand the current disease patterns across the world by describing the magnitude of BCa incidence and mortality and examining their geographic variations according to country, world region, and the four-tier Human Development Index (HDI), where HDI was used to assess the cancer burden at varying levels of development (low, medium, high and very high HDI), using estimates for 2020. These findings will help to better identify populations at higher risk of BCa and provide indications of the causal factors underlying reported geographic variations. Furthermore, we have predicted the future burden of new BCa cases and deaths up to 2040 based on demographic projections with the aim of supporting future planning requirements for optimising the allocation of resources for BCa screening, diagnosis, and therapy.

## METHODS

### Data sources

The number of new cases of, and deaths from, BCa (International Classification of Diseases tenth revision (ICD-10) C67) were extracted from the GLOBOCAN 2020 database for 185 countries or territories, by sex and 5-year age group (0-4, 5-9, …, 80-84, 85 and over) [1,2,18]. GLOBOCAN 2020 database are available from the Global Cancer Observatory [2] which includes facilities for the tabulation and graphical visualisation of the GLOBOCAN database, including explorations of the current burden in 2020 and the future burden by 2040 for 36 cancers (as well as all cancers combined) by sex and by 18 age groups (0-4, 5-9, …, 80-84, 85 and over) in 185 countries or territories. The GLOBOCAN 2020 database, collated by the International Agency for Research on Cancer, comprises national cancer incidence and mortality estimates derived from the best available recorded data available from national (or subnational) cancer registries and national vital registry systems worldwide [1,2,18]. Briefly, cancer incidence estimates were derived from national or subnational population-based cancer registry data including submissions to the Cancer Incidence in Five Continents (volume XI) from 2008 to 2012, and more recent data from the African Cancer Registry Network [1,2,18]. Cancer mortality estimates were obtained using the most recent national vital registration data from World Health Organization (WHO) [1,2,18]. The data sources and methods used in compiling the global cancer estimates for 2020 have been described in detail elsewhere [18]. The population data from the year 2020 to 2040 were retrieved from the United Nations (UN) website [19].

### Statistical analysis

We present tables and figures of the estimated new cases and deaths, as well as two summary measures using direct standardisation, namely the age-standardised incidence rates (ASIRs) and age-standardised mortality rates (ASMRs) per 100 000 person-years based on the 1966 Segi-Doll World standard population [20,21] and the cumulative risk of being diagnosed with or dying from BCa before the age of 75 expressed as a percentage, assuming the absence of competing causes of death [22].

We predicted the future number of BCa cases and deaths worldwide and by the HDI groups up to the year 2040, based on demographic projections and scenarios of annually increasing (+1, +2, +3, +4%), stable (0%) or decreasing (-1, -2, -3, -4%) rates from the baseline year of 2020. We did not use scenarios of rates changing by ±5% or more because such changes would be unlikely to occur in real life. Predictions were calculated by applying the age-specific rates for the year 2020 (and each of the increasing or decreasing scenarios described) to the corresponding projected population data as estimated by the United Nations Development Programme.

The results are presented by country and aggregated across 20 UN-defined world regions [19] and six WHO regions and according to HDI in 2020 [23]. The estimated numbers of cases and deaths have been rounded to three significant figures to avoid spurious precision. In some cases, this creates small discrepancies with the displayed totals and percentages, which are based on the data before rounding. Data management and analyses were performed in R software (version 4.0.2) [24]. Figures were plotted using SigmaPlot software (version 12.5) [25]. Global maps of BCa incidence and mortality rates by country were retrieved from the Global Cancer Observatory, Cancer Today website [2]. Global maps of BCa incidence and mortality rates by country were depicted by ourselves using R software (version 4.0.2) [24] according to World Health Organization data [2].

## RESULTS

### Global burden of bladder cancer incidence and mortality

In 2020, an estimated 573 000 people were diagnosed with BCa worldwide, corresponding to an ASIR of 5.6 per 100 000 (**Table 1**). More men than women were diagnosed with BCa and the ASIRs were approximately 4-fold higher in men than women (Table S1 in the **Online Supplementary Document**). Globally, an estimated 213 000 people died from BCa, corresponding to an ASMR of 1.9 per 100 000 (**Table 1**). Mortality was also higher among men than women (Table S1 in the **Online Supplementary Document**). In addition, the cumulative risk of being diagnosed with and dying from BCa before the age of 75 was one in 53 and one in 112, respectively (**Table 1**).

**Table 1:** Bladder cancer incidence and mortality in both sexes combined in 2020 by world region and human development index level

Region	Population	Incidence				Mortality			
	**No. (%), in thousands**	**Number of cases**	**Percentage of world total**	**ASIR (95% CI)**	Cumulative risk*	**Number of deaths**	**Percentage of world total**	**ASMR (95% CI)**	Cumulative risk*
**Europe**									
Northern Europe	106 261 (1.4)	23 333	4.1	8.7 (8.6-8.9)	2.87	9563	4.5	2.7 (2.7-2.8)	1.39
Western Europe	196 146 (2.5)	68 143	11.9	13.0 (13.0-13.2)	3.92	20 866	9.8	3.0 (3.0-3.0)	1.40
Southern Europe	153 423 (2.0)	61 480	10.7	15.3 (15.3-15.6)	4.29	17 931	8.4	3.3 (3.2-3.4)	1.48
Central and Eastern Europe	293 013 (3.8)	51 027	8.9	8.6 (8.5-8.7)	2.20	18 929	8.9	2.8 (2.7-2.8)	1.00
**America**									
Northern America	368 870 (4.7)	89 997	15.7	10.9 (10.8-10.9)	3.59	21 045	9.9	2.1 (2.1-2.2)	1.04
South America	430 760 (5.5)	27 159	4.7	4.8 (4.8-4.9)	1.58	10 108	4.8	1.6 (1.6-1.7)	0.73
Central America	179 670 (2.3)	4119	0.7	2.2 (2.1-2.3)	0.68	1690	0.8	0.8 (0.8-0.9)	0.36
Caribbean	43 532 (0.6)	2562	0.4	4.1 (3.9-4.2)	1.36	1302	0.6	1.8 (1.8-2.0)	0.82
**Asia**									
Eastern Asia	1 678 090 (21.5)	132 316	23.1	4.3 (4.3-4.4)	1.46	54 206	25.5	1.6 (1.6-1.6)	0.81
South-Central Asia	2 014 709 (25.8)	35 949	6.3	1.9 (1.9-2.0)	0.51	18 243	8.6	1.0 (1.0-1.0)	0.34
South-Eastern Asia	668 620 (8.6)	18 911	3.3	2.6 (2.5-2.60)	0.85	10 327	4.9	1.4 (1.3-1.4)	0.65
Western Asia	278 429 (3.6)	20 915	3.6	8.9 (8.8-9.1)	2.38	7834	3.7	3.2 (3.1-3.2)	1.31
**Oceania**									
Australia and New Zealand	30 322 (0.4)	3923	0.7	5.5 (5.3-5.6)	2.12	1615	0.8	1.8 (1.7-1.9)	1.05
Melanesia	11 123 (0.1)	201	<0.1	2.8 (2.4-3.3)	0.79	104	<0.1	1.6 (1.3-1.9)	0.56
Micronesia/Polynesia	1233 (<0.1)	47	<0.1	3.8 (2.4-5.1)	0.47	26	<0.1	2.1 (1.1-3.2)	0.28
**Africa**									
Northern Africa	246 233 (3.2)	18 589	3.2	8.9 (8.8-9.0)	2.52	10 787	5.1	5.2 (5.1-5.3)	2.17
Western Africa	401 861 (5.2)	4139	0.7	2.0 (1.9-2.0)	0.49	2397	1.1	1.3 (1.2-1.3)	0.37
Southern Africa	67 504 (0.9)	2208	0.4	4.1 (3.9-4.3)	1.28	853	0.4	1.6 (1.5-1.7)	0.59
Middle Africa	179 595 (2.3)	1366	0.2	1.6 (1.5-1.7)	0.47	784	0.4	1.0 (0.9-1.0)	0.35
Eastern Africa	445 406 (5.7)	6894	1.2	3.2 (3.1-3.2)	0.94	3926	1.8	1.9 (1.8-1.9)	0.71
**HDI**									
Very high HDI	1 564 286 (20.1)	356 601	62.2	10.2 (10.2-10.2)	3.13	108 713	51.2	2.5 (2.5-2.5)	1.15
High HDI	2 909 468 (37.3)	164 626	28.7	4.1 (4.1-4.1)	1.26	75 297	35.4	1.8 (1.8-1.8)	0.81
Medium HDI	2 327 556 (29.9)	38 712	6.8	1.9 (1.9-1.9)	0.49	20 719	9.8	1.0 (1.0-1.0)	0.33
Low HDI	990 175(12.7)	13 025	2.3	2.6 (2.5-2.6)	0.76	7693	3.6	1.7 (1.7-1.8)	0.61
**WHO Region**									
WHO Africa	1 124 061 (14.4)	17 526	3.1	3.1 (3.0-3.1)	0.93	9655	4.5	1.8 (1.8-1.8)	0.69
WHO Americas	1 024 551 (13.1)	123 837	21.6	7.3 (7.3-7.4)	2.52	34 145	16.1	1.8 (1.8-1.9)	0.86
WHO East Mediterranean	764 753 (9.8)	31 204	5.4	5.9 (5.9-6.0)	1.65	16 468	7.8	3.2 (3.2-3.3)	1.31
WHO Europe	929 738 (11.9)	221 298	38.6	11.0 (11.0-11.1)	3.22	73 363	34.5	3.0 (3.0-3.0)	1.30
WHO South-East Asia	2 040 083 (26.1)	39 118	6.8	2.0 (2.0-2.0)	0.57	20 890	9.8	1.0 (1.0-1.0)	0.40
WHO Western Pacific	1 928 975 (24.7)	140 171	24.5	4.2 (4.2-4.3)	1.41	57 965	27.3	1.6 (1.6-1.6)	0.79
World	7 794 799 (100)	573 278	100.0	5.6 (5.6-5.6)	1.88	212 536	100.0	1.9 (1.9-1.9)	0.89

ASIR – age-standardised incidence rate per 100 000 person-years, CI – confidence interval, ASMR – age-standardised mortality rate per 100 000 person-years, HDI – Human Development Index, WHO – World Health Organization

*Cumulative risk of being diagnosed with or dying from bladder cancer until 74 years of age in 2020.

### Geographical variation in bladder cancer incidence and mortality by world region

**Figure 1:**
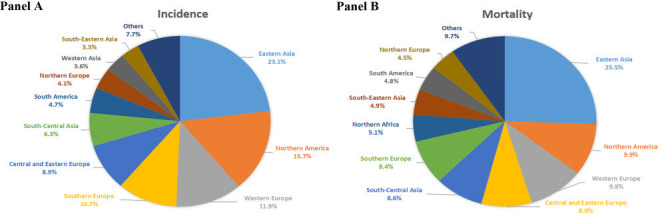
**Panel A.** Distribution of bladder cancer cases for both sexes combined by world region in 2020. **Panel B.** Distribution of bladder cancer deaths for both sexes combined by world region in 2020.

The greatest number of cases and deaths of BCa in both sexes combined were observed in Eastern Asia, followed by Northern America and Western Europe (**Figure 1**, panel A and panel B). Among men and women separately, the top three world regions with the largest number of both BCa cases and deaths remained Eastern Asia, Northern America, and Western Europe (Figure S1 and Figure S2 in the **Online Supplementary Document**). A predominance in men in BCa cases and deaths was consistent across world regions (Table S1 in the **Online Supplementary Document**).

**Figure 2:**
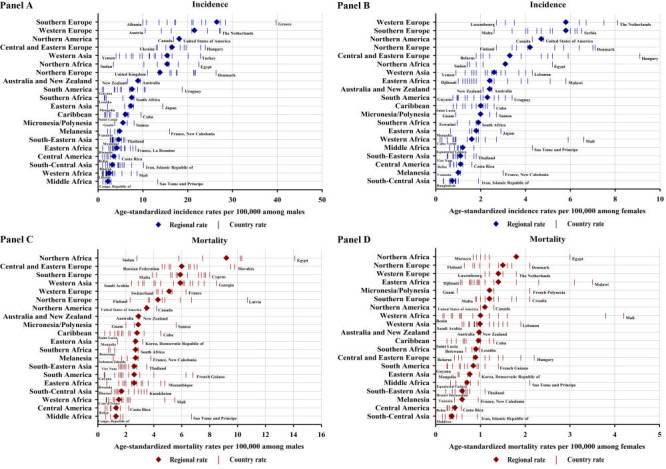
**Panel A.** Age-standardised incidence rates of bladder cancer in men by country within world region. **Panel B.** Age-standardised incidence rates of bladder cancer in women by country within world region. **Panel C.** Age-standardised mortality rates of bladder cancer in men by country within world region. **Panel D.** Age-standardised mortality rates of bladder cancer in women by country within world region. Ordered according to descending rates by world region.

The incidence rates of BCa showed approximately 12-fold variation in men and 8-fold in women across world regions (**Figure 2**, panel A and panel B and Table S1 in the **Online Supplementary Document**). In men, the ASIRs were lowest in Middle Africa but the highest in Southern Europe, followed by Western Europe and Northern America. In women, the ASIRs were lowest in South-Central Asia but the highest in Western Europe, Southern Europe, and Northern America. Between world regions, the mortality rates of BCa varied approximately 7-fold in men and 5-fold in women (**Figure 2**, panel C and panel D and Table S1 in the **Online Supplementary Document**). In men, the highest ASMRs were detected in Northern Africa, followed by Central and Eastern Europe, Southern Europe, and Western Asia, and the lowest were in Middle Africa and Central America. In women, the highest ASMRs were found in Northern Africa, Northern Europe, Western Europe, and Eastern Africa, and the lowest were in South-Central Asia and Central America. Sex-related disparities were also noted, with BCa ASIRs and ASMRs higher in men than women across world regions. For example, the ASIR ratio of men to women ranged from 1.6 in Western Africa to 5.9 in Western Asia, and the ASMR ratio of men to women ranged from 1.5 in Western Africa to 6.7 in Central and Eastern Europe.

By WHO region, the highest ASIR in both sexes combined was detected in Europe, while the highest ASMR was found in East Mediterranean (**Table 1**); the same was true in women (Table S1 in the **Online Supplementary Document**). However, the highest both ASIR and ASMR were observed in Europe (Table S1 in the **Online Supplementary Document**).

### Geographical variations in bladder cancer incidence and mortality by country

**Figure 3:**
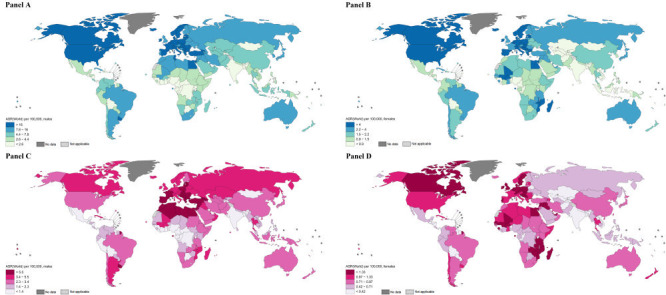
**Panel A.** Global map of age-standardised rates (ASR) of bladder cancer incidence per 100 000 person-years in men by country. **Panel B.** Global map of ASR of bladder cancer incidence per 100 000 person-years in women by country. **Panel C.** Global map of ASR of bladder cancer mortality per 100 000 person-years in men by country. **Panel D.** Global map of ASR of bladder cancer mortality per 100 000 person-years in women by country [2,24].

At the national level, the highest ASIRs occurred in Greece, Italy, Spain, and the Netherlands in men and in Hungary, the Netherlands, and Germany in women (**Figure 2**, panel A and panel B and **Figure 3**, panel A and panel B). Notably, incidence rates varied within world regions, for example, ranging from 0.9 per 100 000 in Guyana to 18.8 in Uruguay in men within South America (high-to-low ASIR ratio = 20.9) and from 0.2 in Cabo Verde to 6.6 in Mali in women within Western Africa (high-to-low ASIR ratio = 33.0) (**Figure 2**, panel A and panel B). The incidence rates were also heterogeneous across countries in high-risk world regions, for example, ranging from 10.0 per 100 000 in Albania to 39.7 in Greece in men within Southern Europe (high-to-low ASIR ratio = 4.0) and from 2.7 in Luxembourg to 8.1 in the Netherlands in women within Western Europe (high-to-low ASIR ratio = 3.0). In terms of mortality, the highest ASMRs were observed in Egypt, Latvia, and Tunisia in men and in Mali, Burkina Faso, and Malawi in women (**Figure 2**, panel C and panel D and **Figure 3**, panel C and panel D). Considerable variations in BCa mortality were also evident within world regions. For example, the ASMRs in South America in men were as low as 0.5 per 100 000 in Guyana and Bolivia and as high as 6.8 in French Guiana (high-to-low ASMR ratio = 13.6) and the ASMRs in Western Africa in women were as low as 0.2 in Benin and Cabo Verde and as high as 4.2 in Mali (high-to-low ASMR ratio = 21.0) (**Figure 2**, panel C and panel D).

### Bladder cancer incidence and mortality by level of human development

By HDI group, the vast majority of BCa cases and deaths occurred among the 57.4% of the world population living in high and very high HDI countries, representing 90.9% of new cases and 86.6% of deaths globally (**Table 1**). A gradient across HDI categories was observed for both BCa incidence and mortality. Bladder cancer incidence rates were substantially higher in countries with very high HDI and high HDI than those with medium and low HDI, while mortality rates were slightly higher in countries with very high HDI and high HDI than those with medium and low HDI (**Table 1**).

### Predicted number and percentage increase of cases and deaths from bladder cancer

**Figure 4:**
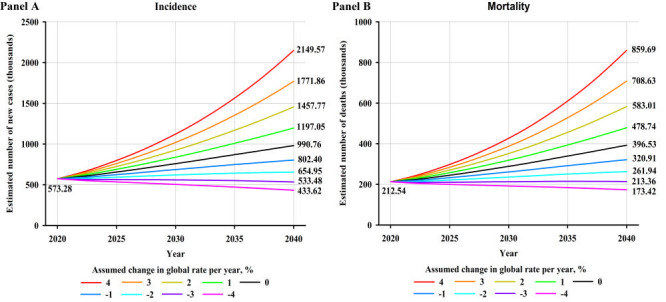
**Panel A.** Predicted number of new bladder cancer cases for both sexes combined assuming nine scenarios of annual change in global incidence rates between 2020 and 2040. **Panel B.** Predicted number of bladder cancer deaths for both sexes combined assuming nine scenarios of annual change in global mortality rates between 2020 and 2040.

Worldwide, the number of new BCa cases were estimated to increase by approximately 72.8%, from 573 000 in 2020 to 991 000 in 2040, assuming global incidence rates in 2020 will remain constant (**Figure 4**, panel A). Moreover, a 1% annual increase in incidence rates from 2020 would more than double the total annual BCa cases by 2040. Notably, an annual decline of 3% in incidence rates would be required to achieve fewer BCa cases in the future compared to the estimated cases in 2020. In terms of mortality, the number of BCa deaths were estimated to increase by approximately 86.6%, from 213 000 in 2020 to 397 000 in 2040, assuming mortality rates in 2020 remained unchanged (**Figure 4**, panel B). A 4% annual decrease in mortality rates would be required to ensure that there would be fewer BCa deaths in 2040 than in 2020.

By WHO region, the largest relative increase in new BCa cases and deaths will occur in Western Pacific, with 105.7% more cases and 115.4% more deaths per year by 2040 (Figure S3 and Figure S4 in the **Online Supplementary Document**), assuming the incidence and mortality rates in 2020 remain constant. It would require a 4%, 3%, 4%, 2%, 3%, and 3% annual decrease in incidence rates to achieve fewer BCa cases in 2040 compared to the estimated number of cases in 2020 for Africa, Americas, East Mediterranean, Europe, South-East Asia, and Western Pacific, respectively. Notably, decreases in mortality rates would need to be greater than 4%, 4%, 4%, 2%, 4%, and 4% to retain the predicted BCa deaths in 2040 at the level observed in 2020 for Africa, Americas, East Mediterranean, Europe, South-East Asia, and Western Pacific, respectively.

**Figure 5:**
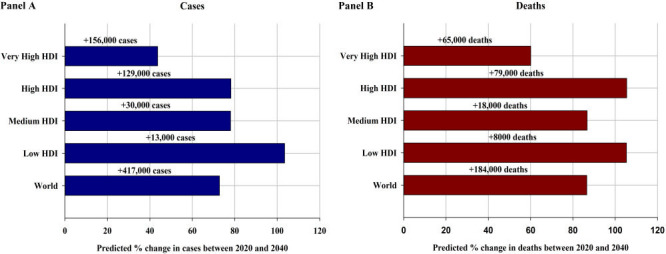
**Panel A.** Predicted percentage change (absolute numbers are shown above bars) of the number of new bladder cancer cases for both sexes combined from between 2020 and 2040, by HDI, assuming incidence rates in 2020 remained unchanged. **Panel B.** Predicted percentage change (absolute numbers are shown above bars) of the number of bladder cancer deaths for both sexes combined from between 2020 and 2040, by HDI, assuming mortality rates in 2020 remained unchanged. HDI – Human Development Index

By HDI group, the largest absolute increase in new BCa cases and deaths will occur in very high HDI countries, with 43.7% more cases and 60.0% more deaths per year by 2040 (**Figure 5**, panel A and panel B), assuming the incidence and mortality rates in 2020 remain constant, reflecting the already high rates in the very high HDI countries and its large population which will continue to grow. However, the greatest relative increases in cases and deaths will occur in low HDI countries (103.5% and 105.3% increases, respectively) and high HDI countries (78.2% and 105.4% increases, respectively). A 1%, 1%, and 2% annual increase in incidence rates from 2020 would nearly double the total annual BCa cases by 2040 for medium, high, and very high HDI countries, respectively (Figure S5 in the **Online Supplementary Document**). Notably, decreases in incidence rates would need to be greater than 4%, 3%, 3%, and 2% to retain the predicted number of new BCa cases in 2040 at the level seen in 2020 for low, medium, high, and very high HDI countries, respectively. It would require a 4%, 4%, 4%, and 3% annual decrease in mortality rates to achieve fewer BCa deaths in 2040 compared to the estimated number of deaths in 2020 for low, medium, high, and very high HDI countries, respectively (Figure S6 in the **Online Supplementary Document**).

## DISCUSSION

Our study identifies high-risk populations for BCa and provides indications for cancer specialists and public health policymakers seeking to plan appropriate BCa control strategies and optimise the allocation of resources accordingly. Specifically, our study found that the higher risk of BCa incidence occurred in Southern and Western European and Northern American populations, while higher risks of mortality were detected in Northern African populations. Regarding sex disparities, men had higher risk than women across all world regions. Furthermore, our study found that BCa cases and deaths will increase by more than 70% over the next 20 years worldwide and highlights the need to develop and implement targeted interventions for high-risk populations to tackle global BCa burden.

We have shed light on the current global distribution of BCa cases and deaths yet the epidemiological profile of BCa burden across regions and countries has been changing. For example, the incidence rates of BCa for both men and women in most European countries have increased and those in most Asian, Oceanian, and South American countries have decreased during 1993-2012 [4]. Hence, the relatively high incidence rates of BCa observed in Europe and the relatively low incidence rates found in Asia, Oceania, and South America could be related to the corresponding ongoing transitions in the epidemiological profile of BCa over the past decade.

The disparities in BCa burden by geography and by sex might be mainly correlated with the different prevalence of its risk factors, such as tobacco smoking, occupational exposure to carcinogenic chemicals, and infection with *Schistosoma haematobium*. Considering a latency period of approximately 30 years from the initiation of smoking to the diagnosis of BCa [3,26], the relatively high incidence rates of BCa observed in European and Northern American countries could be largely attributed to the higher smoking prevalence in these regions in previous decades. For example, the age-standardised smoking prevalence (%) in men in 1980 and 1996 was as high as 54.7 and 53.8 in Greece, 44.3 and 32.2 in Italy, 44.4 and 41.0 in Spain, 34.5 and 29.9 in the Netherlands, and 33.2 and 23.1 in the United States, and in women was 27.4 and 24.1 in Hungary, 25.9 and 25.3 in the Netherlands, 27.8 and 23.2 in Germany, 39.3 and 34.9 in Denmark, 28.3 and 20.1 in the United States, respectively [27]. The higher BCa incidence in men than in women could be largely explained by the tobacco epidemic among women lagging behind that of men by several decades, and the public health effects of the epidemic have yet to mature in women [27].

The relatively elevated BCa incidence in high and very high HDI countries could also possibly be linked to higher rates of occupational exposure to carcinogens in these countries a few decades ago, such as the aromatic amines in the dye industry in the European Union [28]. The relatively high BCa burden in Northern Africa and Western Asia are more likely to be attributable to the higher population-level prevalence (more than 50%) of *Schistosoma haematobium* infection in these regions [29,30]. Furthermore, Northern African and Western Asian countries show between a 5- and 6-fold higher BCa risk in men than women; this finding could be interpreted as a result of the transmission of *Schistosoma haematobium* via agricultural activities typically undertaken by men [13]. Notably, compared with low and medium HDI countries, high and very high HDI countries have a higher proportion of the elderly in their populations [31], resulting in a higher burden of BCa which is more commonly diagnosed in older age [13,17]. Additionally, people in such countries have higher health awareness and can more easily obtain access to advanced diagnostic methods, such as urine cytology, cystoscopy, and computed tomography scan [32,33], which promotes the diagnosis of new BCa cases. However, the diagnostic opportunities and awareness could be the source of error since the HDI may not entirely capture the same point in the national health care development. Thus, the findings presented according to HDI should be interpreted with caution. In terms of BCa mortality, its geographical disparities could be largely explained by the differences in terms of health care systems, clinical practice, and access to diagnosis and treatment facilities between countries at different levels of development of resource levels [3,34].

We suggest that BCa incidence could be largely reduced if the prevalence of modifiable risk factors was reduced to a minimum in regions with larger population attributable fractions (PAFs). The PAF for BCa incidence due to tobacco smoking was estimated to be 39.5% in men and 22.6% in women in European countries, with the largest PAF being 45.3% in men in Central and Eastern European countries and 35.2% in women in Northern European countries [35]. The PAF for BCa incidence due to occupational exposure to carcinogens was estimated to be 10.6% in men and 11.4% in women in East Asian countries [36]. The PAF for the incidence of squamous cell carcinoma of the bladder due to *Schistosoma haematobium* infection was estimated to be 100% in most African and Western Asian countries [37]. With such a high contribution of modifiable risk factors to BCa incidence in certain regions, research to estimate the impact of changes in major risk factors on the future incidence of BCa would provide useful insight for BCa prevention strategies in varying contexts.

A previous study showed that the increases of new BCa cases from 2006-2016 worldwide were mainly due to population growth and ageing [38]. In our study, the projected number of cases and deaths from BCa are expected to increase to 981 000 and 392 000 by 2040 worldwide, respectively, as a result of population growth and aging alone. A 3% annual decrease in global incidence rates and a 4% annual decline in global mortality rates would be needed to halt the increasing BCa burden by 2040. Considering these changes, the reallocation of resources for BCa primary prevention programmes aimed at reducing population levels of tobacco smoking, occupational exposure to carcinogenic chemicals, and *Schistosoma haematobium* infection in certain regions and countries and the increased access to early detection modalities and health care services for high-risk populations identified by our study are crucial for reducing the global BCa burden.

The numbers and rates of BCa presented in this study are estimates based on the best available data (reviewed for their completeness, coverage and accuracy) from population-based cancer registries. Our study provides a comprehensive picture of the epidemiological profile of BCa incidence and mortality on a global scale in 2020, with an emphasis on region-, country- and sex-specific disparities as well as differences between HDI groups, which is highly relevant for cancer control and clinical practice. Furthermore, our study predicts the future BCa incidence and mortality worldwide and by HDI group up to the year 2040 based on scenarios of rates changing from -4 to 4% from the baseline year of 2020. These predictions might help optimise the allocation of resources for screening, diagnosis, and therapy and provide a benchmark for evaluating BCa prevention and control interventions. Nevertheless, several limitations should be addressed. First, although our findings are based on the best available and high-quality data, caution is warranted when interpreting the findings for countries with limited coverage from population-based cancer registries and where proxy data were used to obtain national estimates [18]. Second, the GLOBOCAN estimates did not account for the impact of the coronavirus 2019 (COVID-19) pandemic on cancer diagnoses because the GLOBOCAN estimates were based on extrapolations of previous years of cancer data [18]. Third, the projections of the future burden of BCa in 2040 neither considered recent changes in BCa incidence and mortality rates nor heterogeneity in BCa incidence and mortality trends between countries. Thus, the predictions in our study likely represent a conservative estimate (an underestimation) of the future BCa burden and should be interpreted with caution. Finally, our study was unable to distinguish the required changes in modifiable risk factors and their corresponding effects on predicted BCa incidence and mortality rates which suggests the need for further studies to clarify the impact of changes in exposure to modifiable risk factors on the burden of BCa.

## CONCLUSIONS

Bladder cancer continues to be a considerable public health challenge worldwide. Distinct variation exists in BCa incidence and mortality across world regions and countries, possibly related to differences in tobacco smoking, occupational exposure to carcinogenic chemicals, and *Schistosoma haematobium* infection. The global share of both BCa cases and deaths was disproportionally high in high and very high HDI countries compared to low and medium HDI countries. Given the considerable geographic disparity of BCa burden across world regions and countries and the corresponding ongoing transition in its epidemiological profile, this study provides new insights into global BCa burden. Considering the predicted 73 and 87% increase in annual BCa cases and deaths by 2040 globally, respectively, this study also highlights the urgent need to promote the development of BCa control strategies and to prioritise allocation of BCa prevention and treatment resources for high-risk populations to tackle the global burden of BCa and narrow its geographical disparities.

## Additional material


Online Supplementary Document
