# Supplementary material for: The global landscape of bladder cancer incidence and mortality in 2020 and projections to 2040
Source: J Glob Health. 2023 Sep 15;13:04109. doi: 10.7189/jogh.13.04109 (PMC10502766; doi:10.7189/jogh.13.04109)
Supplement: Online Supplementary Document [file jogh-13-04109-s001.pdf]

Table S1. Bladder cancer incidence and mortality in 2020 by sex, world region, and human development index level

Region	Incidence							Mortality						
	Males			Females			M:F rate ratio	Males			Females			M:F rate ratio
	Cases, No. (%)	ASIR (95% CI)	Cum. Risk*	Cases, No. (%)	ASIR (95% CI)	Cum. Risk*		Deaths, No. (%)	ASMR (95% CI)	Cum. Risk*	Deaths, No. (%)	ASMR (95% CI)	Cum. Risk*	
Europe														
Northern Europe	17,151 (3.9)	13.8 (13.6-14.0)	4.77	6182 (4.7)	4.2 (4.1-4.3)	1.39	3.3	6678 (4.2)	4.3 (4.2-4.4)	2.32	2885 (5.4)	1.5 (1.5-1.6)	0.73	2.9
Western Europe	51,854 (11.8)	21.5 (21.3-21.7)	6.90	16,289 (12.3)	5.8 (5.7-5.9)	1.69	3.7	15,341 (9.7)	5.1 (5.1-5.2)	2.55	5525 (10.3)	1.4 (1.3-1.4)	0.64	3.6
Southern Europe	48,889 (11.1)	26.5 (26.2-26.8)	7.85	12,591 (9.5)	5.8 (5.7-6.0)	1.58	4.6	14,000 (8.8)	5.9 (5.8-6.0)	2.85	3931 (7.3)	1.2 (1.2-1.3)	0.56	4.9
Central and Eastern Europe	38,764 (8.8)	16.5 (16.4-16.7)	4.67	12,263 (9.3)	3.3 (3.3-3.4)	0.90	5.0	14,797 (9.3)	6.0 (5.9-6.1)	2.37	4132 (7.7)	0.9 (0.9-0.9)	0.36	6.7
America														
Northern America	69,080 (15.7)	18.1 (18.0-18.2)	6.29	20,917 (15.8)	4.7 (4.6-4.8)	1.52	3.9	15,129 (9.5)	3.5 (3.4-3.5)	1.80	5916 (11.0)	1.1 (1.1-1.1)	0.51	3.2
South America	19,618 (4.4)	7.6 (7.5-7.7)	2.75	7541 (5.7)	2.3 (2.2-2.4)	0.77	3.3	7067 (4.5)	2.6 (2.5-2.7)	1.26	3041 (5.7)	0.8 (0.8-0.8)	0.38	3.1
Central America	2963 (0.7)	3.5 (3.3-3.6)	1.11	1156 (0.9)	1.1 (1.1-1.2)	0.34	3.2	1207 (0.8)	1.3 (1.2-1.4)	0.58	483 (0.9)	0.4 (0.4-0.5)	0.18	3.0
Caribbean	1830 (0.4)	6.1 (5.8-6.4)	2.18	732 (0.6)	2.0 (1.8-2.2)	0.71	3.1	917 (0.6)	2.8 (2.6-3.0)	1.32	385 (0.7)	1.0 (0.9-1.1)	0.44	2.9
Asia														
Eastern Asia	102,408 (23.2)	7.3 (7.2-7.3)	2.59	29,908 (22.6)	1.8 (1.8-1.8)	0.63	4.1	39,717 (25.0)	2.7 (2.6-2.7)	1.41	14,489 (27.0)	0.8 (0.8-0.8)	0.40	3.6
South-Central Asia	29,000 (6.6)	3.2 (3.2-3.2)	0.88	6949 (5.2)	0.7 (0.7-0.7)	0.18	4.4	14,838 (9.3)	1.7 (1.6-1.7)	0.58	3405 (6.3)	0.4 (0.4-0.4)	0.13	4.7
South-Eastern Asia	14,620 (3.3)	4.6 (4.5-4.7)	1.56	4291 (3.2)	1.1 (1.1-1.1)	0.36	4.2	7857 (4.9)	2.6 (2.5-2.7)	1.22	2470 (4.6)	0.6 (0.5-0.6)	0.29	4.4
Western Asia	17,498 (4.0)	15.4 (15.1-15.7)	4.49	3417 (2.6)	2.6 (2.5-2.7)	0.77	5.9	6472 (4.1)	5.9 (5.7-6.1)	2.57	1362 (2.5)	1.0 (1.0-1.1)	0.44	6.0
Oceania														
Australia and New Zealand	2995 (0.7)	8.9 (8.6-9.2)	3.56	928 (0.7)	2.4 (2.2-2.6)	0.94	3.7	1160 (0.7)	2.9 (2.7-3.1)	1.75	455 (0.8)	1.0 (0.9-1.1)	0.52	3.0
Melanesia	163	4.8	1.34	38	1.0	0.31	4.8	85	2.7	0.94	19	0.6	0.24	4.6

	(<0.1)	(4.5-5.1)		(<0.1)	(0.6-1.4)			(0.1)	(2.1-3.3)		(<0.1)	(0.3-0.9)		
Micronesia/Polynesia	35 (<0.1)	5.6 (3.2-7.9)	0.70	12 (<0.1)	2.0 (0.7-3.3)	0.24	2.8	18 (<0.1)	2.9 (1.2-4.6)	0.38	8 (<0.1)	1.2 (0.2-2.2)	0.05	2.4
Africa														
Northern Africa	15,131 (3.4)	15.4 (15.1-15.7)	4.45	3458 (2.6)	3.1 (3.0-3.2)	0.96	5.0	8769 (5.5)	9.2 (9.0-9.4)	3.98	2018 (3.8)	1.8 (1.7-1.9)	0.81	5.1
Western Africa	2399 (0.5)	2.5 (2.4-2.6)	0.63	1740 (1.3)	1.6 (1.5-1.6)	0.38	1.6	1386 (0.9)	1.5 (1.4-1.6)	0.46	1011 (1.9)	1.0 (0.9-1.1)	0.29	1.5
Southern Africa	1605 (0.4)	7.5 (7.1-7.9)	2.73	603 (0.5)	1.9 (1.8-2.0)	0.53	3.9	566 (0.4)	2.7 (2.5-3.0)	1.11	287 (0.5)	0.9 (0.8-1.0)	0.33	3.0
Middle Africa	841 (0.2)	2.2 (2.1-2.3)	0.64	525 (0.4)	1.2 (1.1-1.3)	0.34	1.8	483 (0.3)	1.3 (1.1-1.4)	0.48	301 (0.6)	0.7 (0.6-0.7)	0.26	1.9
Eastern Africa	4020 (0.9)	4.2 (4.0-4.3)	1.38	2874 (2.2)	2.4 (2.3-2.4)	0.64	1.8	2298 (1.4)	2.6 (2.5-2.7)	1.07	1628 (3.0)	1.4 (1.3-1.5)	0.48	1.9
HDI														
Very high HDI	275,369 (62.5)	17.5 (17.5-17.6)	5.74	81,232 (61.6)	4.2 (4.2-4.2)	1.28	4.2	80,336 (50.6)	4.4 (4.4-4.4)	2.15	28,377 (53.5)	1.1 (1.1-1.1)	0.52	4.0
High HDI	126,996 (28.8)	6.8 (6.7-6.8)	2.19	37,630 (28.5)	1.7 (1.7-1.7)	0.56	4.0	57,133 (36.0)	3.1 (3.1-3.1)	1.44	18,164 (34.2)	0.8 (0.8-0.8)	0.37	4.0
Medium HDI	30,178 (6.8)	3.0 (3.0-3.0)	0.83	8534 (6.5)	0.8 (0.8-0.8)	0.20	3.8	16,414 (10.3)	1.7 (1.7-1.7)	0.57	4305 (8.1)	0.4 (0.4-0.4)	0.13	4.3
Low HDI	8076 (1.8)	3.6 (3.5-3.7)	1.10	4459 (3.4)	0.7 (0.7-0.7)	0.16	5.5	4812 (3.0)	2.3 (2.2-2.4)	0.91	2209 (4.2)	0.3 (0.3-0.3)	0.12	7.0
WHO Region														
WHO Africa	11,397 (2.6)	4.5 (4.4-4.6)	1.47	6129 (4.6)	1.9 (1.8-1.9)	0.54	2.4	6218 (3.9)	2.6 (2.5-2.7)	1.1	3437 (6.4)	1.1 (1.0-1.1)	0.4	2.4
WHO Americas	93,491 (21.2)	12.3 (12.2-12.4)	4.44	30,346 (22.9)	3.3 (3.3-3.4)	1.1	3.7	24,320 (15.3)	2.9 (2.9-2.9)	1.49	9825 (18.3)	0.9 (0.9-1.0)	0.43	3.2
WHO East Mediterranean	25,385 (5.8)	9.8 (9.7-9.9)	2.75	5819 (4.4)	2.2 (2.1-2.3)	0.63	4.5	13,299 (8.4)	5.4 (5.3-5.5)	2.17	3169 (5.9)	1.2 (1.1-1.2)	0.53	4.5
WHO Europe	171,347 (38.9)	19.7 (19.6-19.8)	6.12	49,951 (37.7)	4.3 (4.3-4.3)	1.28	4.6	55,912 (35.2)	5.6 (5.6-5.7)	2.58	17451 (32.5)	1.1 (1.1-1.2)	0.52	5.1
WHO South-East Asia	30,665 (7.0)	3.2 (3.2-3.2)	0.97	8453 (6.4)	0.8 (0.8-0.8)	0.24	4.0	16,485 (10.4)	1.8 (1.8-1.8)	0.69	4405 (8.2)	0.4 (0.4-0.4)	0.17	4.3
WHO Western Pacific	108,478 (24.6)	7.0 (7.0-7.1)	2.51	31,693 (23.9)	1.7 (1.7-1.7)	0.61	4.1	42,510 (26.8)	2.7 (2.7-2.7)	1.38	15455 (28.8)	0.7 (0.7-0.7)	0.39	3.7
World	440,864	9.5	3.36	132,414	2.3	0.80	4.0	158,785	3.3	1.60	53,751	0.9	0.41	3.8

(100)	(9.5-9.5)	(100)	(2.3-2.4)	(100)	(3.3-3.3)	(100)	(0.9-0.9)

ASIR – age-standardized incidence rate per 100,000 person-years, CI – confidence interval, ASMR – age-standardized mortality rate per 100,000 person-years, HDI – Human Development Index, WHO – World Health Organization

* Cumulative risk of being diagnosed with or dying from bladder cancer until 74 years of age in 2020.

Figure S1

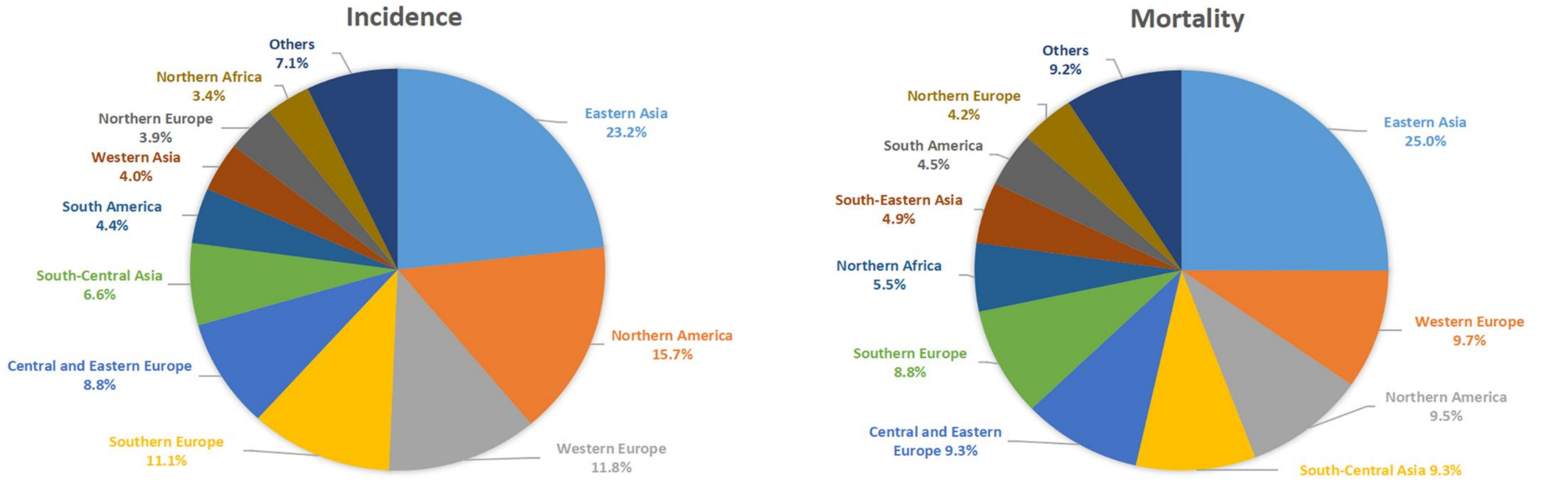

Figure S2

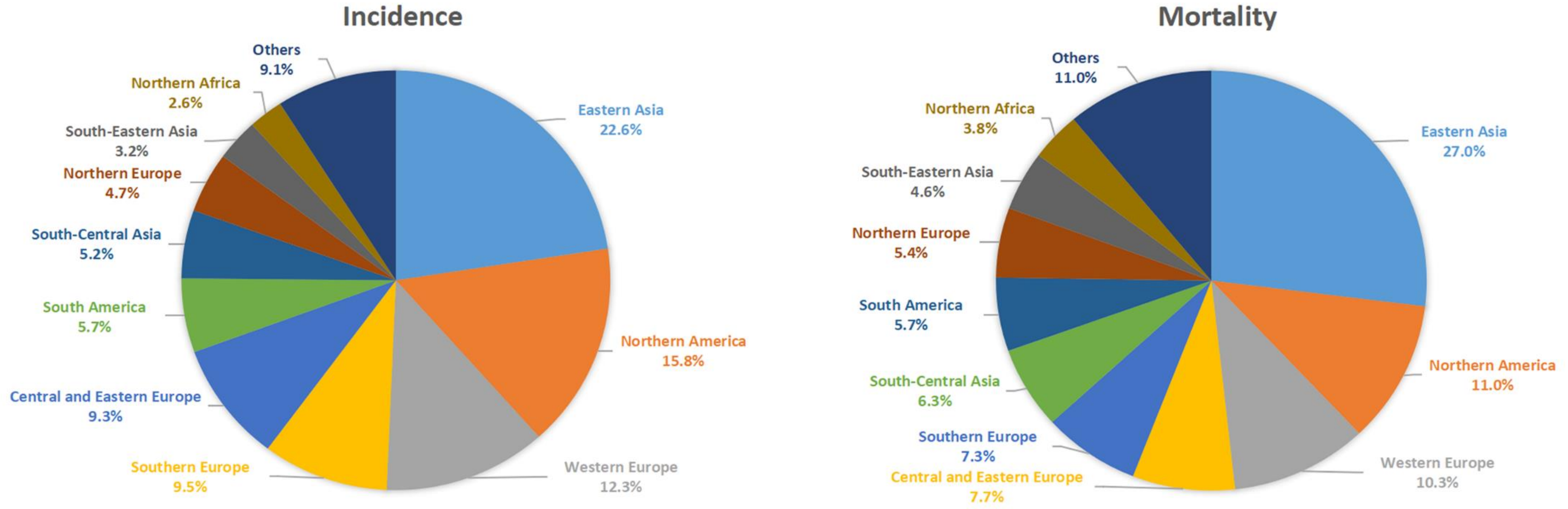

Figure S3

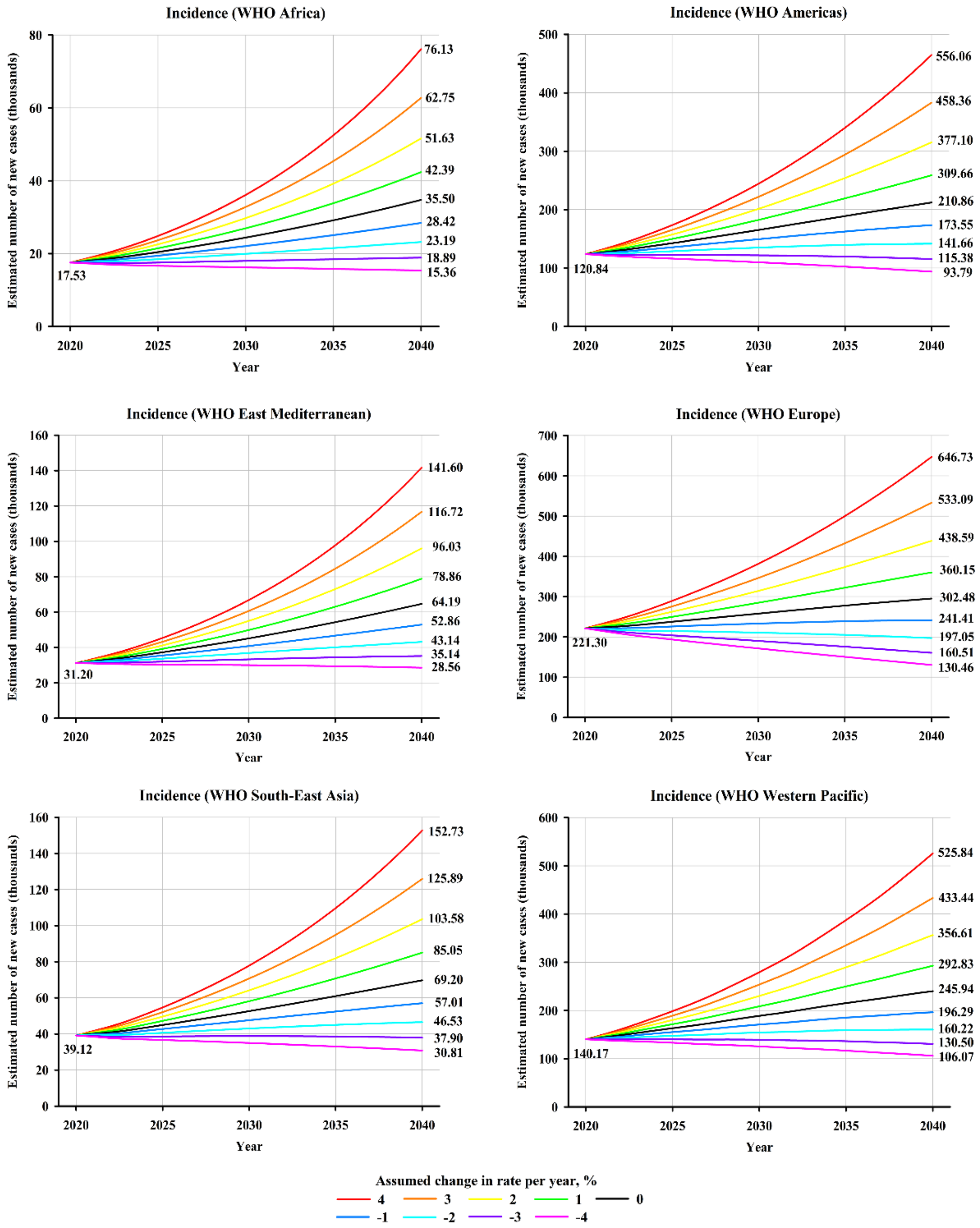
Incidence (WHO Africa)
Estimated number of new cases (thousands)
Year
17.53
76.13
62.75
51.63
42.39
35.50
28.42
23.19
18.89
15.36
Incidence (WHO Americas)
Estimated number of new cases (thousands)
Year
120.84
556.06
458.36
377.10
309.66
210.86
173.55
141.66
115.38
93.79
Incidence (WHO East Mediterranean)
Estimated number of new cases (thousands)
Year
31.20
141.60
116.72
96.03
78.86
64.19
52.86
43.14
35.14
28.56
Incidence (WHO Europe)
Estimated number of new cases (thousands)
Year
221.30
646.73
533.09
438.59
360.15
302.48
241.41
197.05
160.51
130.46
Incidence (WHO South-East Asia)
Estimated number of new cases (thousands)
Year
39.12
152.73
125.89
103.58
85.05
69.20
57.01
46.53
37.90
30.81
Incidence (WHO Western Pacific)
Estimated number of new cases (thousands)
Year
140.17
525.84
433.44
356.61
292.83
245.94
196.29
160.22
130.50
106.07
Assumed change in rate per year, %
4
3
2
1
0
-1
-2
-3
-4

Figure S4

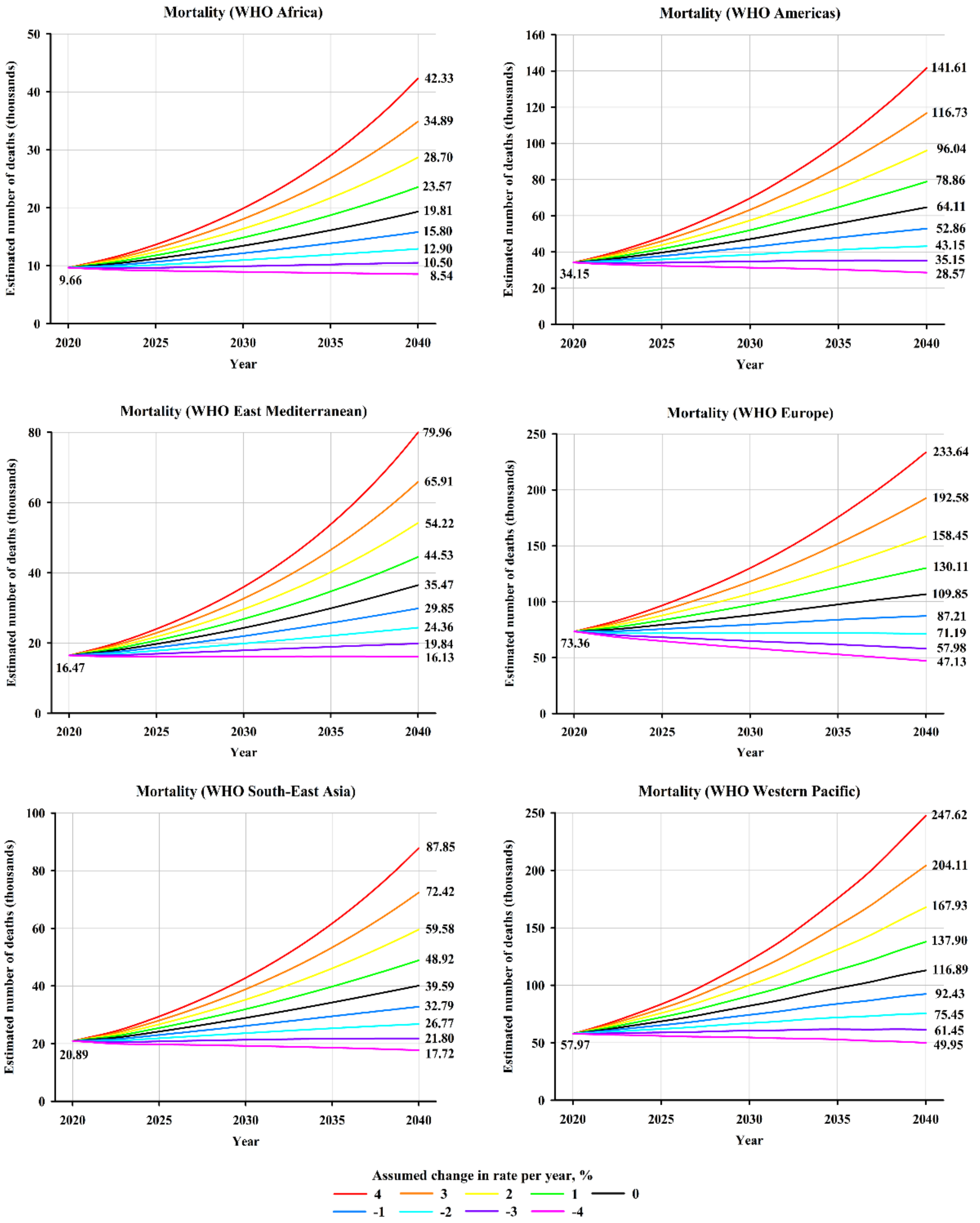
Mortality (WHO Africa)
Estimated number of deaths (thousands)
Year
9.66
42.33
34.89
28.70
23.57
19.81
15.80
12.90
10.50
8.54
Mortality (WHO Americas)
Estimated number of deaths (thousands)
Year
34.15
141.61
116.73
96.04
78.86
64.11
52.86
43.15
35.15
28.57
Mortality (WHO East Mediterranean)
Estimated number of deaths (thousands)
Year
16.47
79.96
65.91
54.22
44.53
35.47
29.85
24.36
19.84
16.13
Mortality (WHO Europe)
Estimated number of deaths (thousands)
Year
73.36
233.64
192.58
158.45
130.11
109.85
87.21
71.19
57.98
47.13
Mortality (WHO South-East Asia)
Estimated number of deaths (thousands)
Year
20.89
87.85
72.42
59.58
48.92
39.59
32.79
26.77
21.80
17.72
Mortality (WHO Western Pacific)
Estimated number of deaths (thousands)
Year
57.97
247.62
204.11
167.93
137.90
116.89
92.43
75.45
61.45
49.95
Assumed change in rate per year, %
4 3 2 1 0
-1 -2 -3 -4

Figure S5

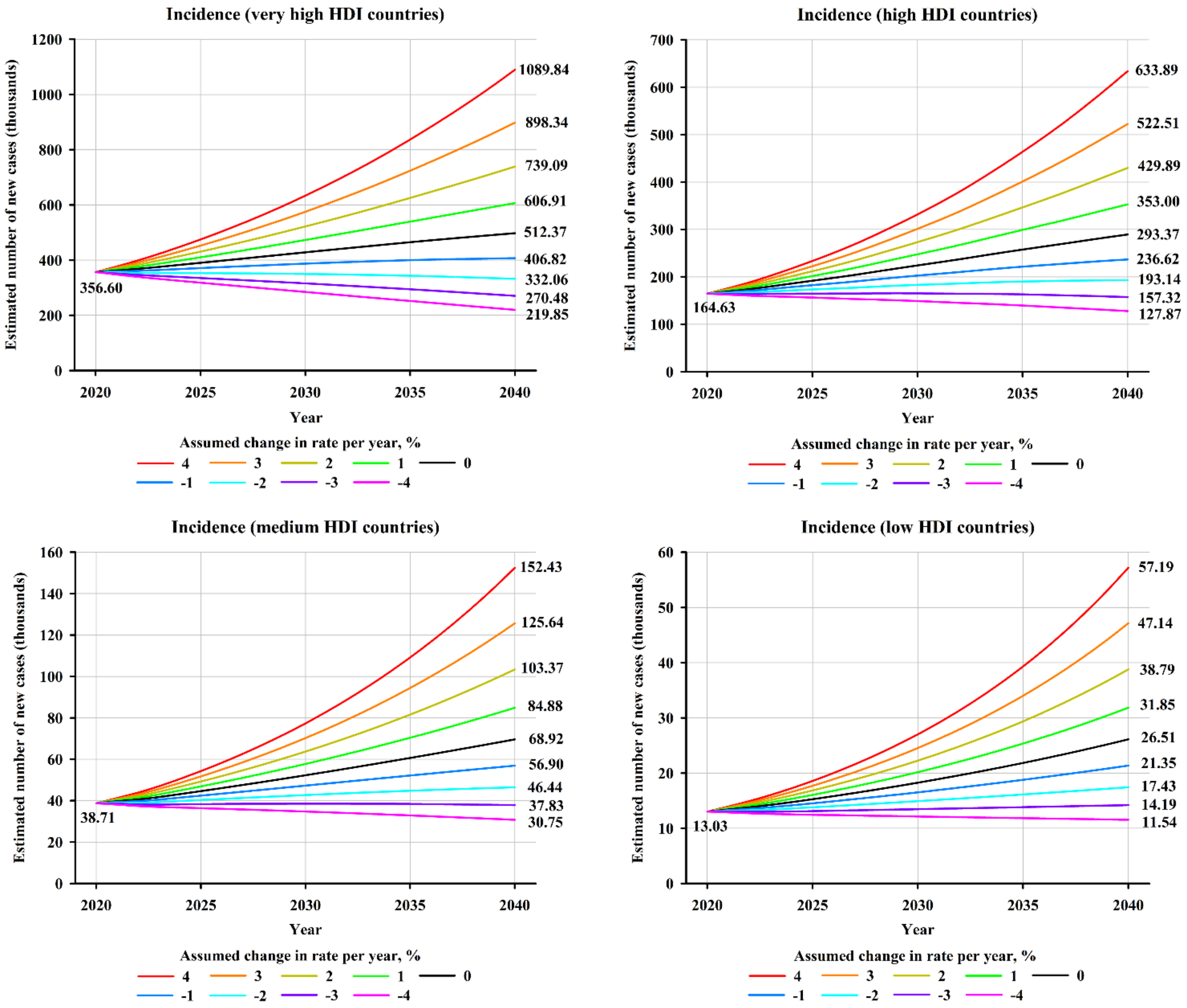
Incidence (very high HDI countries)
Estimated number of new cases (thousands)
Year
356.60
1089.84
898.34
739.09
606.91
512.37
406.82
332.06
270.48
219.85
Assumed change in rate per year, %
4
3
2
1
0
-1
-2
-3
-4
Incidence (high HDI countries)
Estimated number of new cases (thousands)
Year
164.63
633.89
522.51
429.89
353.00
293.37
236.62
193.14
157.32
127.87
Assumed change in rate per year, %
Incidence (medium HDI countries)
Estimated number of new cases (thousands)
Year
38.71
152.43
125.64
103.37
84.88
68.92
56.90
46.44
37.83
30.75
Assumed change in rate per year, %
Incidence (low HDI countries)
Estimated number of new cases (thousands)
Year
13.03
57.19
47.14
38.79
31.85
26.51
21.35
17.43
14.19
11.54
Assumed change in rate per year, %

Figure S6

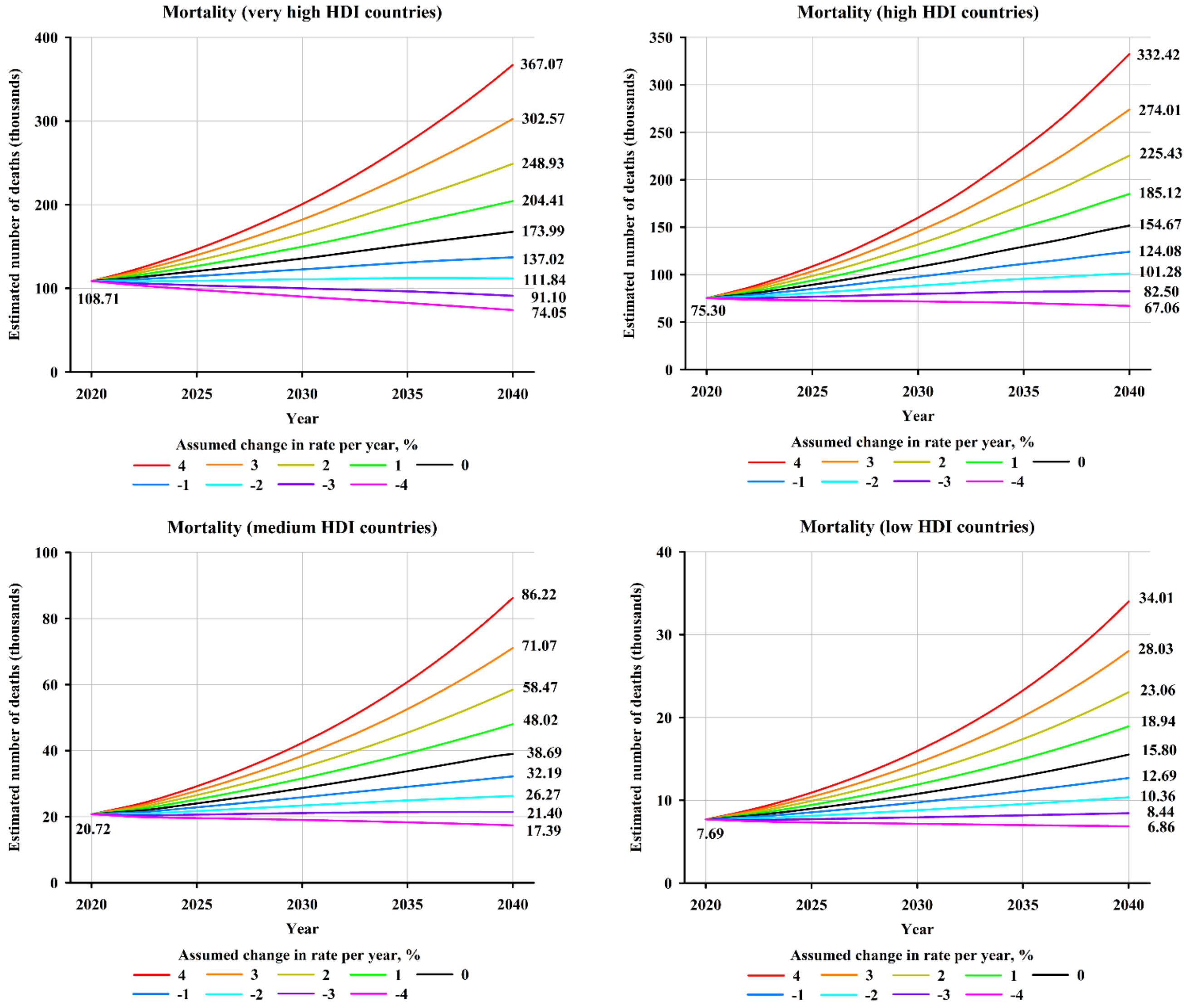
Mortality (very high HDI countries)
Estimated number of deaths (thousands)
400
300
200
100
0
2020
2025
2030
2035
2040
Year
367.07
302.57
248.93
204.41
173.99
137.02
111.84
91.10
74.05
108.71
Assumed change in rate per year, %
4
3
2
1
0
-1
-2
-3
-4
Mortality (high HDI countries)
Estimated number of deaths (thousands)
350
300
250
200
150
100
50
0
2020
2025
2030
2035
2040
Year
332.42
274.01
225.43
185.12
154.67
124.08
101.28
82.50
67.06
75.30
Assumed change in rate per year, %
4
3
2
1
0
-1
-2
-3
-4
Mortality (medium HDI countries)
Estimated number of deaths (thousands)
100
80
60
40
20
0
2020
2025
2030
2035
2040
Year
86.22
71.07
58.47
48.02
38.69
32.19
26.27
21.40
17.39
20.72
Assumed change in rate per year, %
4
3
2
1
0
-1
-2
-3
-4
Mortality (low HDI countries)
Estimated number of deaths (thousands)
40
30
20
10
0
2020
2025
2030
2035
2040
Year
34.01
28.03
23.06
18.94
15.80
12.69
10.36
8.44
6.86
7.69
Assumed change in rate per year, %
4
3
2
1
0
-1
-2
-3
-4
